# Effects of a prehabilitation program on patients’ recovery following spinal stenosis surgery: study protocol for a randomized controlled trial

**DOI:** 10.1186/s13063-015-1009-2

**Published:** 2015-10-27

**Authors:** Andrée-Anne Marchand, Margaux Suitner, Julie O’Shaughnessy, Claude-Édouard Châtillon, Vincent Cantin, Martin Descarreaux

**Affiliations:** Université du Québec à Trois-Rivières, Trois-Rivières, Québec Canada; Centre de Santé et de Services Sociaux de Trois-Rivières, Trois-Rivières, Québec Canada

**Keywords:** Prehabilitation, Exercise therapy, Lumbar spine, Spinal stenosis, Surgery

## Abstract

**Background:**

Degenerative lumbar spinal stenosis is a prevalent condition in adults over the age of 65 and often leads to deconditioning. Although the benefits of surgery outweigh those of conservative approaches, physical rehabilitation may be used to improve function and to minimize the risk of persistent dysfunction. This study protocol was designed to establish the feasibility of a full-scale randomized controlled trial and to assess the efficacy of an active preoperative intervention program on the improvement of clinical parameters and functional physical capacity in patients undergoing surgery for lumbar spinal stenosis.

**Methods/Design:**

Forty patients will be recruited and randomly allocated to one of the 2 treatment arms: 6 weeks supervised preoperative rehabilitation program (experimental group) or hospital standard preoperative management (control group). The intervention group will be trained three times per week, with each session aiming to improve strength, muscular endurance, spinal stabilization and cardiovascular fitness. Intensity and complexity of exercises will be gradually increased throughout the sessions, depending on each participant’s individual progress. Primary outcomes are level of low back disability and level of pain. Secondary outcomes include the use of pain medication, quality of life, patient’s global impression of change, lumbar extensor muscles endurance, maximum voluntary contraction of lumbar flexor and extensor muscles, maximum voluntary contraction of knee extensors, active lumbar ranges of motion, walking abilities, and cardiovascular capacity. Both the primary and secondary outcomes will be measured at baseline, at the end of the training program (6 weeks after baseline evaluation for control participants), and at 6 weeks, 3 and 6 months postoperatively.

**Discussion:**

This study will inform the design of a future large-scale trial. Improvements of physical performances before undergoing lumbar surgery may limit functional limitations occurring after a surgical intervention. Results of this study will provide opportunity to efficiently improve spinal care and advance our knowledge of favorable preoperative strategies to optimize postoperative recovery.

**Trial registration:**

US National Institutes of Health Clinical Trials registry NCT02258672, 10 February 2014.

## Background

Lumbar spinal stenosis (LSS) is one of the most frequent degenerative conditions in older-aged patients [1] and the most common indication for spine surgery in patients older than 65 years [2]. Although several treatment approaches have been suggested in the literature, there is an increasing body of evidence in favor of surgery-related treatment for the management of LSS [3]. For instance, recent evidence shows that decompressive laminectomy/laminotomy alone or combined with fusion for LSS outweighs the effects of non-surgical interventions [3]. In spite of advances in surgical techniques, outcomes following spine surgery are highly variable and advantages following spinal stenosis surgery seem to diminish over time with outcomes becoming similar to those of conservative management at 5 years postoperatively [4]. Furthermore, the frequency of spinal stenosis complex surgeries has increased in the recent years and has been associated with increased costs, risks of complications and resource use, and lower success rates [5, 6].

Although the benefits of surgery outweigh those of conservative approaches for patients with LSS, physical rehabilitation may be used to improve function and to minimize the risk of persistent dysfunction. In fact, a limited course of active physical therapy is initially recommended for patients with LSS [7]. Such a decision may be motivated by the fact that watchful waiting of LSS does not result in catastrophic progressions of neurologic deficits [3]. In addition, there is low-quality evidence suggesting that exercise therapy leads to better short-term outcomes than no exercise with respect to disability, back and leg pain [8]. However, the current scientific knowledge does not provide sufficient evidence to establish clear recommendation for or against the use of physical therapy or exercise as stand-alone treatments for degenerative LSS [7]. Beyond the effectiveness as stand-alone treatments lies the potential adjuvant effects of conservative modalities to surgical outcomes, which are, however, not clearly understood.

On the other hand, the effects of postoperative rehabilitation following lumbar surgery have been extensively studied and yielded optimistic perspectives to improve patients’ recovery. A recent systematic review investigated whether active rehabilitation programs following primary surgery for LSS have an impact on functional outcomes and whether such programs are superior to “usual” postoperative care [9]. The study suggests that active rehabilitation is more effective than usual care in improving both short-term and long-term functional status and back and leg pain in adults who underwent spinal decompressive surgery (with or without fusion) for the first time [9].

Although improvements in pain and disability following spine rehabilitation programs have been described in the early postoperative phase, preoperative muscle dysfunctions such as decreased trunk muscle strength and imbalances between trunk extensor and flexor muscles have been reported up to 3 months after surgical fusion [10]. Furthermore, surgical interventions and techniques are known to entail varying levels of invasiveness which can objectively be measured by the levels of biochemical markers of systemic inflammatory response and muscle damage [11]. Such changes will potentially affect muscle function and recovery following surgery. Overall, these results suggest that surgery mostly decreases pain and gross disability but does not systematically lead to improved muscle conditioning. Characterization of postoperative physical capacity deficits should inform the development of targeted approaches aimed at developing improved pain coping and pain-avoidance mechanisms, whilst simultaneously improving overall physical fitness and function.

Authors do acknowledge that much debate persists on which surgical approach is the most cost-effective and may also offer favorable long-term outcomes [2]. Development of innovative surgical techniques, combined with efforts to optimize perioperative patients’ management have increased the focus on improvement of postoperative outcomes. The “fast-track surgery” multimodal approach, which focuses on enhancing recovery and reducing morbidity by optimizing preoperative and postoperative patient management, has shown to effectively enhance recovery and reduce need for hospitalization while keeping readmission rates unchanged [12, 13]. Similarly, patient activation, defined as one’s propensity to engage in positive health, has been identified as an important modifier of the recovery process, leading to better outcomes and increased compliance to physical therapy after lumbar surgery [14]. Santa Mina et al. recently conducted a systematic review investigating the effectiveness of total-body and region-specific prehabilitation interventions for patients awaiting hip or knee arthroplasty, amongst other conditions [13]. The authors reported improved physical function at the preoperative assessment and improved physical function, length of hospital stay, pain and quality of life following surgery, compared with standard care. We conducted a scoping review exploring current practices in perioperative rehabilitation for lumbar spine surgery and identified only one randomized control trial investigating the effects of a preadaptation program. The study, by Nielsen et al., compared the impact of an integrative program of care, combining both preadaptation and early postoperative rehabilitation, to routine procedures for patients undergoing elective surgery for degenerative disease [15]. Despite the reported improvements in postoperative function, recovery and hospital stay, the pragmatic design of the study does not allow to tease out the effectiveness of individual interventions.

The Nielson study results, coupled with the positive preliminary results, reported in favor of rehabilitation programs before knee and hip arthroplasty [16–19] has triggered the following question: would the implementation of a preoperative rehabilitation protocol specifically designed for the lumbar region improve the outcomes of surgical interventions? Consequently, the main purpose of the proposed research protocol is to determine the feasibility of conducting a full-scale, randomized controlled trial of prehabilitation versus usual care, and to estimate treatment effects on the improvement of patients’ postoperative clinical and functional status.

## Methods/Design

### Design

This study is a randomized controlled pilot clinical trial. It is design to assess the feasibility and efficacy of an active preadaptation programme in improving patients’ recovery following a LSS surgery. The primary outcomes are level of low back disability (as measured by the Oswestry Disability Index (ODI) version 2.1a) and level of pain as measured by a Numerical Rating Scale (NRS)). The proposed study protocol has been approved by the institutional review boards of the *Université du Québec à Trois-Rivières* and *Centre de Santé et de Services Sociaux de Trois-Rivières* (CÉR-2014-008-00), where the study will take place. The trial has been registered with the US National Institutes of Health Clinical Trials registry (NCT02258672). Informed written consent will be obtained from each participant before any intervention is initiated.

### Recruitment

Participants will be recruited at the *Centre de Santé et de Services Sociaux de Trois-Rivières* during their encounter with their treating neurosurgeon once the decision has been made to move forward with a surgical intervention. If deemed eligible, patients willing to participate in the study will be provided with an overview of the study purpose and content and they will be asked permission to be contacted by the research team. Further details will be given over the phone by a member of the research team and an assessment date will be set for the participant. Participants will be guided through the informed consent process and baseline demographics and physical measures will be collected. At the end of the initial assessment visit, a member of the research team will open the sealed envelope to randomly allocate participants to one of the two groups (intervention versus control). Treatments will be schedule after randomization for participants in the intervention group.

### Eligibility criteria

The inclusion criteria for study subjects are: having a clinical history and diagnostic imaging evidence of LSS, being over 18 years, having degenerative LSS affecting one or multiple vertebral levels, awaiting LSS surgery (minimally invasive or open approach), and being able to provide written informed consent voluntarily. The exclusion criteria are: non-degenerative LSS, presence of inflammatory arthritic conditions, altered cognitive capacities, and vertebral instability requiring non-instrumental or instrumented fusion, and individuals deemed ineligible by their treating neurosurgeon and those unable to comprehend or express themselves in French.

### Sample size

Determination of the sample size was guided by time constraints as well as by a thorough analysis of the patients’ surgery rate conducted over a 1-year period at the recruitment site in accordance with our inclusion and exclusion criteria [20]. Furthermore, when one of the study goals is to obtain an estimate of variance in an outcome when an important difference between groups has already been identified (meaning that only the variance needs to be estimated), it is suggested that 10 to 20 participants per group is deemed sufficient to inform feasibility and to plan for a larger study [20].

### Randomization

Treatment allocation will be made based on randomization and minimization methods. Minimization criteria have been decided based on factors identified by the neurosurgeons known to delay postoperative recovery and include presence of diabetes, objective motor deficits in the lower limbs (confirmed by electromyography), self-reported severe disability (ODI score ≥ 41 %) and smoking habit. Patients will be randomized following a computer- generated list of random numbers. An independent research assistant blinded to patient assignment will sequentially number the envelopes containing intervention assignments according to the computer-generated randomization. Opaque and sealed envelopes will be opened in front of the participants at the end of the initial assessment visit.

### Interventions

Patients will be randomly allocated to one of the two following groups: (1): an intervention group (preoperative supervised physical training) and (2) a control group (hospital standard preoperative management). The intervention group will be trained 3 times per week for 6 weeks prior to their surgery.

### Preoperative physical training

**Fig. 1:**
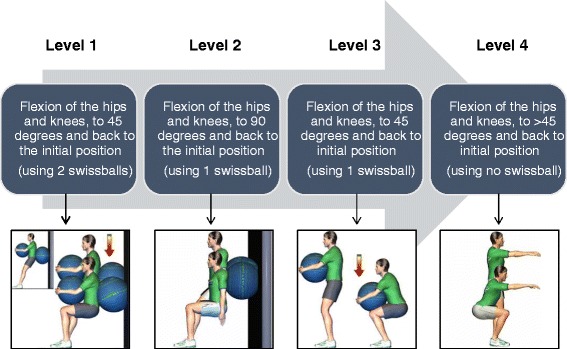
Progression of squat exercise

The preoperative physical training will be supervised by a chiropractor and a certified kinesiologist. Each 30-minute training session will aim to improve strength, muscular endurance and spinal stabilization and will be divided into 3 phases. The first phase consists of a 5-minute cardiovascular warm-up (stationary bicycle). The second consists of three exercises (squat, superman, legs raise) designed to increased stabilization of the spine achieved by co-contracting the abdominal and back muscles. The third phase involves two exercises (hips raise, hips abduction) to strengthen the thigh and hip muscles. The training protocol has been developed so that each specific exercise can be modified to obtain three different levels of difficulty in order to provide a safe and individualized training experience for each participant. Intensity and complexity of the exercises will be gradually increased throughout the sessions, depending on each participant’s individual progress. Our choice of intervention was informed by a scoping literature review of the nature and effectiveness of currently used interventions for patients undergoing any type (excluding scoliosis) of low back surgery (work submitted for publication). Figure 1 illustrates a typical example of the possible modifications for the proposed exercises.

### Hospital preoperative management

Patients undergoing surgical intervention at the regional hospital do not receive any particular physical intervention (exercises, manual therapy, etc.). On their last encounter before the day of surgery they are given a pamphlet summarizing tips on how to keep a good back posture when getting in or out of bed and when sitting down. All participants will be questioned on the amount and type of physical activity performed weekly, if any, regardless of their group allocation.

### Data collection

This study will take into consideration the evaluation of both clinical parameters (pain intensity, lumbar disability, use of pain medication, quality of life) and physical functional capacities (endurance and strength of lumbar region muscles, lumbar ranges of motion, walking abilities, cardiovascular capacity). All physical measures will be obtained during in-clinic assessments; at baseline, at the end of the training program (6 weeks after baseline evaluation for control participants), and at 6 weeks postoperatively and will provide insight into why the prehabilitation program was successful or not. In addition, self-reported questionnaires will be mailed at 3 and 6 months postoperatively in order to provide a subjective evaluation of patients’ recovery at mid-term and long-term. The timeline illustrating the various interventions and outcome assessments is presented in Table 1.

**Table 1:** Schedule of interventions and outcome measures

Time point	Location	Data collection
Baseline assessment	In clinic (UQTR)	Questionnaires^a^
		Physical measurements
6 weeks prehabilitation	In clinic (UQTR)	Physical fitness evolution
Post-intervention, Preoperative assessment	In clinic (UQTR)	Questionnaires
		Physical measurements
Surgery	Hospital (CSSSTR)	Perioperative data^b^
6-week follow-up	In clinic (UQTR)	Questionnaires
		Physical measurements
3-month follow-up	Mailing	Questionnaires^c^
6-month follow-up	Mailing	Questionnaires^c^

^a^Questionnaires include NRS, ODI, EQ-5D-3 L, Tampa Scale of Kinesiophobia, and BDI

^b^Perioperative data refers to blood loss, length of surgery and intraoperative complications

^c^In addition to the regular questionnaire packet, a home-made questionnaire regarding physical activities undertaken during the postoperative phase will be mailed to the participants

*BDI* Beck Disability Index, *CSSSTR Centre de Santé et de Services Sociaux de Trois-Rivières*, *EQ-5D-3 L* EuroQol-5D-3 L, *ODI* Oswestry Disability Index, *NRS* Numerical Rating Scale, * UQTR Université du Québec à Trois-Rivières*

### Measures of feasibility

Full-scale trial feasibility will be assessed by recruitment rate (and reasons for non-participation) and attrition rate, adherence to the protocol and safety of the intervention. Adherence to the prehabilitation protocol will be monitored through the kinesiologist’s logbook. For every participant, date of encounter and exercises performed (including, number of repetitions and levels of difficulty, perceived effort and discomfort – location, intensity and character) will be recorded. Exercises have been specifically designed as to not aggravate stenotic symptoms (slight lumbar flexion is preserved) while performing them. However, in case of unusual or worsening discomfort, the chiropractor will re-evaluate the participant to ensure that there are no safety issues or possible threats to the participant. If we encounter a number of problems with a given exercise, even with its easiest version, the specific exercise will be considered irrelevant for the population under investigation and the research team will discuss how to come up with an alternative that targets the same objective.

### Primary outcome measures

#### Back and leg pain

Pain intensity will be assessed using an 11-point NRS. Each patient will subjectively rate their current level of leg and back pain on a scale of 0 to 10 (0 being an absence of pain 10 being the worst pain imaginable) [21].

#### Back disability

Disability related to back pain will be measured via the validated French version of the ODI questionnaire (version 2.1a) [22]. The ODI contains ten questions related to daily activities, including pain intensity, personal care, lifting, walking, sitting, standing, sleeping, sexual life, social life, and traveling. Each question is rated on a scale of 0 to 5 points with a maximum of 50. Higher scores indicate greater disability.

### Secondary outcome measures

#### Pain medication

The use of pain medication will be evaluated as the number of pills taken in a day using the patient’s hospital chart for the hospital stay period and via a daily self-reported journal for the preoperative and postoperative at-home period.

#### Quality of life

The quality of life will be measured using the EuroQol-5D-3 L questionnaire (EQ-5D-3 L). The EQ-5D-3 L comprises the following 5 dimensions: mobility, self-care, usual activities, pain/discomfort and anxiety/depression. Each dimension has three levels: no problems, some problems, extreme problems. Scores obtained on the EQ-5D-3 L should not be considered as a cardinal score but as an index of perceived health-related quality of life [23].

#### Global impression of change

The patient’s global impression of change will be measured using the Clinical Global Impression - Improvement scale (CGI-I). The 7-point scale ranging from 1: very much improved to 7: very much worse, will be used to evaluate patient’s perception of change in his or her condition over the preoperative 6-week period. Global impression of change will be measured at the preoperative assessment only and will help evaluate the prehabilitation program effects.

#### Lumbar extensor muscles endurance

Trunk muscle endurance assessment will be done with a modified version of the Sorenson test using an inclined bench. Participants will be positioned on a 30° Roman chair, the iliac crest aligned with the chair’s border, the upper body maintained in a horizontal position (parallel to the floor) and the arms crossed on the chest. The test will be stopped when the participant is no longer able to maintain a proper horizontal position (as evaluated by the researcher), becomes too fatigued to continue, or experiences pain.

#### Maximum voluntary contraction

Strength of knee extensor muscles will be assessed using a load cell (Model LSB350; Futek Advanced Sensor Technology Inc., Irvine, CA, USA). Strength of lumbar flexor and extensor muscles will be assessed using an isokinetic testing device (LIDO, Loredan Biomedical Inc., Davis, CA, USA). Each measurement will be taken three times and the mean value will be used for subsequent analyses.

#### Lumbar ranges of motion

Active lumbar ranges of motion will be assessed with an inclinometer (Digital Dualer IQ Pro™ Digital Inclinometer, Model CM101; JTECH Medical, Midvale, UT, USA). Each range of movement will be measured twice and the mean value will be used for analysis.

#### Walking abilities

Walking abilities will be assessed using two components of the exercise treadmill examination (time to first symptoms (TFS) and total ambulation time (TAT)). Both variables will be measured at a walking speed of 1.2 mph, on a 4 ° inclined ramp. If ever a participant is unable to keep up with the predetermined walking speed, the examination will be performed using the participant’s preferred walking speed. The examination will be stopped at the onset of severe symptoms, defined as the level of discomfort that would cause the patient to stop walking in usual life situations. Walking abilities will serve as an indicator of surgical success.

#### Maximal aerobic capacity

The maximal aerobic capacity will be estimated based on the results obtained during a submaximal aerobic test and using a linear extrapolation method. The submaximal aerobic test is performed with a Monark ergocycle (Ergomedic 828-E Test Bike, Monark, Varberg, Sweden) and consists of a 3-minute warm-up with an initial workload (from 0.25 kp to 0.5 kp) successively followed by 3-minute periods with progressively increasing workloads until an 85 % maximal heart rate is reached. Time to 85 % maximal heart rate will be recorded.

### Additional measurements

Participants will complete a series of questionnaires and clinical examination procedures during the baseline assessment, allowing for exploratory analysis of any baseline features that might be predictors of intervention response. The questionnaires will include the following: demographics including age, gender, months lived with pain, presence of comorbidities and conservative interventions received so far. The Tampa Scale of Kinesiophobia will be used at every time assessment point to evaluate fear of movement and of re-injury because it has been found to be associated with poor performance on a number of physical tests and high scores have proven to be a powerful predictor of postoperative disability. The Beck Disability Index (BDI) will be used at every assessment time point as a screening tool for possible signs of high levels of anxiety and depression. The Minnesota Satisfaction Questionnaire (MSQ) will be used during the baseline evaluation only in order to assess participant’s satisfaction related to work conditions for those employed or on sick leave at the time of the study. Clinical examination will include weight and height measurements and a neurological exam of lower extremity deep tendon reflexes, assessment of fine touch and pinprick sensation, manual muscle testing for strength, ankle clonus and the Babinski test. In addition, participants from both groups will be asked about nature, length and frequency of any physical activities undertaken during the preoperative (assessed orally at the preoperative encounter) and postoperative periods (mailed questionnaire at 3-month and 6-month follow-up).

### Statistical analysis

Participants’ characteristics will be summarized using descriptive statistics (mean, standard deviation). The equivalence of groups at baseline in terms of demographic and clinical variables will be assessed using independent samples *t* tests for continuous variables and chi-square tests for categorical variables. Following an intention-to-treat principle, estimates of efficacy (Group and Time main effect, as well as Group x Time interactions) will be analyzed using a repeated-measure analysis of variance (ANOVA). The statistical significance level will be set at 5 % and missing data will be analyzed using multiple imputation methods.

### Ethical considerations

Considering that patients are on a waiting list for their surgical intervention, it is possible that the training period will vary from one individual to the next. No intervention presented herein will delay or interfere with the patients’ surgical planning. All participants have the right to withdraw from the study at any time and the research team reserves the right to stop the trial if it is believed that there are risks of adverse events or if the patients’ condition or symptoms worsen.

## Discussion

Despite the development of innovative surgical approaches and efforts being made to optimized treatment plans for the management of low back pain, the role of exercise therapy as an adjunct to operative modalities remains unclear. This pilot study will provide preliminary data that will be used as a ‘stepping stone’ for a future large-scale clinical study investigating the effects of a prehabilitation program on patients’ recovery following spinal stenosis surgery. In addition to the assessment of possible effects and associations that may be worth investigating in a subsequent larger trial, the study will allow for documentation of patient compliance and satisfaction regarding a prehabilitation protocol based on exercise training.

In a broader perspective, the observations gathered during the pilot study will expand the knowledge related to the functional changes affecting patients after lumbar surgery, allowing for identification of specific components of physical fitness that should be targeted in future prehabilitation protocols. Similarly, it is believed that the results of this study could eventually lead to improvement in the delivery of spinal care. Identification of patients’ characteristics likely to respond favorably to prehabilitation will help target the individuals for whom it should be considered to limit symptoms persistence, and post-surgery complications and side-effects.

### Trial status

The trial is currently recruiting participants. Enrollment and trial completion is expected to be finished by the end of 2015.

## Abbreviations

ANOVA: analysis of variance
BDI: Beck Disability Index
CGI-I: Clinical Global Impression - Improvement scale
EQ-5D-3 L: EuroQol-5D-3 L questionnaire
LSS: lumbar spinal stenosis
MSQ: Minnesota Satisfaction Questionnaire
NRS: Numerical Rating Scale
ODI: Oswestry Disability Index
TAT: total ambulation time
TFS: time to first symptoms
